# ML-DTD: Machine Learning-Based Drug Target Discovery for the Potential Treatment of COVID-19

**DOI:** 10.3390/vaccines10101643

**Published:** 2022-09-30

**Authors:** Sovan Saha, Piyali Chatterjee, Anup Kumar Halder, Mita Nasipuri, Subhadip Basu, Dariusz Plewczynski

**Affiliations:** 1 Department of Computer Science & Engineering, Institute of Engineering & Management, Salt Lake Electronics Complex, Kolkata 700091, India; 2 Department of Computer Science & Engineering, Netaji Subhash Engineering College, Techno City, Panchpota, Garia, Kolkata 700152, India; 3 Faculty of Mathematics and Information Sciences, Warsaw University of Technology, Koszykowa 75, 00-662 Warsaw, Poland; 4 Laboratory of Functional and Structural Genomics, Centre of New Technologies, University of Warsaw, Banacha 2c Street, 02-097 Warsaw, Poland; 5 Department of Computer Science & Engineering, Jadavpur University, 188, Raja S.C. Mallick Road, Kolkata 700032, India

**Keywords:** COVID-19 human drug targets, COVID-19 human drug non-targets, machine learning, gene set enrichment analysis, network centrality, protein sequence, docking

## Abstract

Recent research has highlighted that a large section of druggable protein targets in the Human interactome remains unexplored for various diseases. It might lead to the drug repurposing study and help in the in-silico prediction of new drug-human protein target interactions. The same applies to the current pandemic of COVID-19 disease in global health issues. It is highly desirable to identify potential human drug targets for COVID-19 using a machine learning approach since it saves time and labor compared to traditional experimental methods. Structure-based drug discovery where druggability is determined by molecular docking is only appropriate for the protein whose three-dimensional structures are available. With machine learning algorithms, differentiating relevant features for predicting targets and non-targets can be used for the proteins whose 3-D structures are unavailable. In this research, a Machine Learning-based Drug Target Discovery (ML-DTD) approach is proposed where a machine learning model is initially built up and tested on the curated dataset consisting of COVID-19 human drug targets and non-targets formed by using the Therapeutic Target Database (TTD) and human interactome using several classifiers like XGBBoost Classifier, AdaBoost Classifier, Logistic Regression, Support Vector Classification, Decision Tree Classifier, Random Forest Classifier, Naive Bayes Classifier, and K-Nearest Neighbour Classifier (KNN). In this method, protein features include Gene Set Enrichment Analysis (GSEA) ranking, properties derived from the protein sequence, and encoded protein network centrality-based measures. Among all these, XGBBoost, KNN, and Random Forest models are satisfactory and consistent. This model is further used to predict novel COVID-19 human drug targets, which are further validated by target pathway analysis, the emergence of allied repurposed drugs, and their subsequent docking study.

## 1. Introduction

Discovering Drugs or vaccines for the pandemic outbreak of COVID-19 is a time taking process. If it can be done following an exact process, it may take a couple of months to be ready to use as no such effective therapeutic drugs are discovered to combat COVID-19. Moreover, it has several variants produced by continuous variations, and some variants, Delta and Omicron, spread the infection very quickly. In that case, Drug repurposing comes into play, and it may give alternative solutions for therapeutics or medical practitioners to face challenges. As we know, most known drugs are used for repositioning, and they produce promising results. For both cases, druggable target selection through appropriate ranking is significant because selecting appropriate drug targets helps us to determine the proper drug which can be used further in the clinical development and trials for COVID-19 treatment. Many antiviral, antibiotics or other types like Flavipiravir, Remdesivir, Lopinavir, Ritonavir, Azithromycin, and Hydroxy-chloroquine were tried but not able to produce promising results [1]. Drug repurposing requires more understanding of the interaction between pathogens of COVID-19 and host proteins. Identifying host proteins and potential drug targets will greatly facilitate drug repurposing or identification.

Experimental techniques to find druggable targets are affinity pull-downs, pooled RNAi Data [2], and, more recently, genome –CAS9 screens [3], which are always time-consuming and labor-intensive. Comparatively, to deal with the growing number of large-scale human genomics and proteomics datasets, the computational approach is appropriate in terms of cost and time. Structure-based drug discovery [4,5,6] is a kind of computational approach in which protein’s druggability is determined by molecular docking methods [4,5,6] to reveal ideas on macromolecule’s 3D conformation [4,5,6], protein-ligand interactions [4,5,6], and binding affinity of the target proteins [4,5,6]. However, this is limited to proteins whose 3-D Structure is known but will not apply to proteins without having information.

According to Behery et al. [7], computational methods are categorized into ligand, docking, and chemogenomic. Chemogenomic methods are superior to the other two methods in terms of time cost or unavailability of 3-D structure proteins. Chemogenomic methods are classified into machine learning-based, graph-based, and network-based, where machine learning methods have turned out to produce reliable results. This paper used a combination of Physico-chemical features, sequence information, and features derived from Smiles (Simplified Molecular Input Line). They adopted a methodical prediction scheme exploiting ensemble classifiers like LightBoost, XGBoost, ExtraTree, deep learning (DBN), and traditional machine learning methods (random forest), support vector machine.

Another work Wang et al. [8] presented is based on deep learning. They represented drug-target pairs using a scheme where drug molecules are encoded as fingerprint features, and target proteins are represented using their evolutionary information in the form of Legendre moments. Features set thus formed are again reduced by sparse Principal Component Analysis (SPCA). The Deep Long Short-Term Memory (DeepLSTM) model predicts Drug Target Interaction (DTI).

The research work [9], based on Machine learning techniques, proposed a work that can predict druggability scores of protein targets by exploiting distinguishable features from non-targets. In this approach, protein sequence-derived features, protein interaction information, and gene ontology terms are extracted as features, and the model is built on a training dataset. A very recent work based on neural networks on DTI is proposed by Li et al. [10], where 2D paired distance maps of target proteins and molecular graphs of drugs are used. A mutual interaction neural network is designed to capture the interactive impacts between drugs and targets and combine transformed modules by constructing a communicative Message Passing Neural Network (CMPNN).

Another recent work by Barman et al. [1] proposed a network biology approach to identify host targets for discovering drugs by constructing a human protein-protein interaction network (PPIN) with the above SARS-CoV-2 targeted proteins. This work combines essential network centrality measures and functional properties of the human proteins to identify the critical human targets of SARS-CoV-2, namely PRKACA, RHOA, CDK5RAP2, and CEP250.

Adhami et al. [11] presented repurposing novel candidate drugs for COVID-19. This work proposes a network-based drug repurposing strategy where seven potential drugs are identified. First, the PPIN is constructed for 524 human proteins. Then the target miRNAs of the mentioned module genes were separately obtained from the miRWalk 2.0 database because of the vital role of miRNAs in biological processes. Thus, they specified seven clusters of proteins as the complexes of proteins associated with the SARS-CoV-2 virus. Moreover, seven therapeutic candidate drugs were identified to control gene regulation in COVID-19. They are Paclitaxel, Bortezomib, Carboplatin, Crizotinib, Cytarabine, Daunorubicin, and Vorinostat.

Inspired by these works described and considering the current need of the present time, we have hypothesized that druggable COVID-19 human targets can be predicted using GSEA, protein sequence-derived features, and network properties from the PPIN. The host targets for COVID-19 drugs are fetched from TTD (Therapeutic Target Database) [12]. A positive-negative target dataset is constructed by considering approved, clinical and investigational drugs with corresponding indications and their respective targets. Next, ML-DTD is applied to this dataset by a set of Machine learning classifiers to predict COVID-19 human drug targets. Gradually, some novel COVID-19 human drug targets are also identified, from which five COVID-19 repurposed drugs are highlighted.

## 2. Material & Methods

### 2.1. Dataset

A COVID-19 drug target is usually considered a protein indispensably linked with the process of COVID-19 disease progression. Several drugs could handle these targets to produce the desired consequences for the recovery from the pandemic of COVID-19. The Therapeutic Target Database (TTD) [12] is used in this research work on COVID-19 since it involves relevant information for implementing the drug discovery process, which demands both drugs and their associated therapeutic targets [13]. Besides this, 204,961 reviewed human proteins; their respective sequences are also extracted from UniProt [14]. Positive samples comprise ninety COVID-19 human drug targets enlisted in the TTD database. Negative samples are formed of ninety COVID-19 human drug non-targets randomly selected from the 204,961 reviewed human proteins after removing the positive samples.

### 2.2. Workflow of ML-DTD

**Figure 1:**
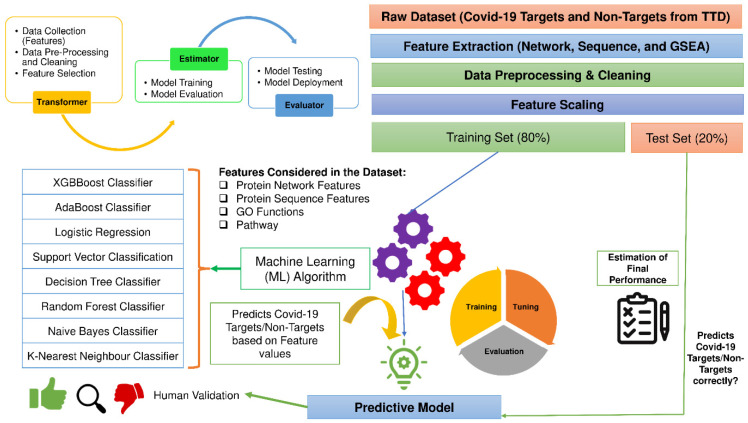
Computational workflow of ML-DTD.

The proposed methodology of ML-DTD consists of the following stages (please see Figure 1): (a) Feature Extraction, (b) Feature Selection, (c) Data Cleaning and Preprocessing, (d) Classification Algorithms, (e) Prediction of novel COVID-19 human drug targets, (f) Drug-Repurposing Study of novel COVID-19 human drug targets and (g) Docking study of the repurposed drugs detected through novel COVID-19 human drug targets. In feature extraction, several characteristics or attributes are fetched for the COVID-19 target and non-target human proteins. Once fetched, feature selection is applied to select the most significant features by filtering out the less important ones. Then the data obtained is preprocessed in the data cleaning and preprocessing stage to filter out the null values and make the data available for processing further. The refined data is inputted into the various machine learning models for learning and predicting by splitting the data into training and test set. These models are discussed in brief in the classification algorithms section. Once the accuracy of the models is fetched, the top performing models are used to predict the novel drug targets in the prediction of novel COVID-19 human drug targets stage. Finally, the related novel drug targets are mapped to their corresponding existing drugs by COVID-19Db in the drug-Repurposing study of novel COVID-19 human drug targets stage. These drugs are validated by docking studies with the other available COVID-19 proteases by DockCoV2 in the last stage, i.e., Docking study of the repurposed drugs detected through novel COVID-19 human drug targets.

(a) Feature Extraction

**Figure 2:**
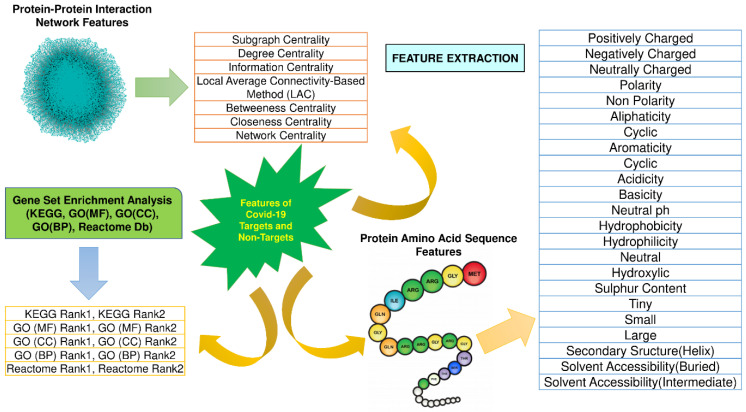
PPIN features Protein Sequence features, and GSEA Ranking features.

To execute Machine Learning algorithms, a pre-defined set of features are needed from the training set to generate the output for the test set. This research initially computed forty feature values for ninety positive and negative samples derived from Protein-Protein Interaction Network (PPIN), protein sequence, and Gene Set Enrichment Analysis (GSEA). More in-depth details of these forty features are highlighted in Figure 2. *Network-based features* like Closeness Centrality [15], Betweenness Centrality [16], etc., are computed by using the CytoNCA app [17] of the Cytoscape [18], where proteins are given as the input. Their corresponding interactions are fetched from the String Db [19]. *Protein Sequence-based features* like Polarity, Non-Polarity, etc., are computed for each amino acid sequence of proteins obtained from UniProt [14] using the web server Pfeature [20]. *GSEA* of proteins have been computed on KEGG [21], Molecular Function (MF) [22], Cellular Component (CC) [22], Biological Process (BP) [22], and Reactome Pathway [23] by using python modules, and the top two ranks in each of them have been included in the feature list.

(b) Feature Selection

Feature selection is an automatic or manual selection of a subset of the most relevant and appropriate features from a set of pre-defined features that can be used to build machine learning models. Out of the initial selection of forty features, the top twenty features are selected based on the ranking provided by the SelectKBest function of the python module sklearn [24]. *SelectKBest* can select k best features based on the type of scoring function the user will provide. In this case, the *chi2 scoring function* is used for this purpose. The detailed ranking and the score of the top twenty selected features are highlighted in Table 1.

(c) Data Cleaning and Preprocessing

Data cleaning and preprocessing is the initial step to transforming the raw data into an understandable format that can be used for further scientific analysis. It can be categorized into the following stages: (1) Gathering the data along with feature values (2) import of the data and inclusion of python library files (3) handling missing (null) values (4) Detection of dependent and independent variables (5) handling of categorical values (if present) (6) Train and Test splitting of data (7) Scaling of feature to normalize them with a fixed range of values. All these steps are followed, and finally, the entire curated dataset with features is split into 80% train and 20% test data to execute machine learning algorithms. This research problem is categorized as a binary classification problem with two labels: Zero and One. *Zero* stands for *COVID-19 human drug non-targets*, and *One* stands for *COVID-19 human drug targets*. The entire working mechanism is highlighted in Figure 1.

(d) Classification Algorithms

Out of the several algorithms, the following eight algorithms are used to predict COVID-19 human drug targets and non-targets: (1) XGBBoost Classifier [25], (2) AdaBoost Classifier [26], (3) Logistic Regression [27], (4) Support Vector Classification [28], (5) Decision Tree Classifier [29], (6) Random Forest Classifier [30], (7) Naive Bayes Classifier [31], and (8) K-Nearest Neighbour Classifier [32].

*Extreme Gradient Boosting* (XGBBoost) is one of the most popular classifiers in recent times. Here the decision trees are sequentially created. Weights have a significant impact on this classifier. All independent variables are allocated some weights, which are then transmitted to the decision trees to predict results. Variable weights wrongly predicted by the decision tree are increased and serve as an input to the second decision tree. Thus, a robust model is ensembled through each type of predictor.

*AdaBoost Classifier* is another methodology of ensemble learning like XGBBoost. It is primarily created to enhance binary classifier efficiency. In this classifier, weak classifiers are turned into stronger ones using weights through an iterative process similar to the XGBBoost classifier.

*Logistic Regression (LR)* is mainly used as a classification algorithm. It leads to estimating the probability of an event occurring based on the independent variables as provided in the dataset. The dependent variable thus gets confined between 0 and 1 since the result is always a probability.

*Support Vector Machine (SVM)* is a popular machine learning algorithm for regression and classification-based problems. The data here are mapped explicitly in a vector space to generate a hyperplane so that *n*-dimensional space can be segregated into classes which can be used to plot a new data point in an appropriate categorization later.

*Decision Tree* comes under the classification of supervised machine learning algorithms. It is mainly known for rule-based decisions. It is a tree-based model where each node represents a test to be performed on the feature, its associated edges signify the decision rules, whereas the leaf nodes denote the possible results of the test.

*Random Forest Classifier (RF)* is one of the easy and flexible machine learning algorithms used in machine learning. Trees form the forest. Forests having significantly more trees are generally more robust. Decision trees are created on random data samples by the random forest. Prediction from each tree is obtained, and the best solution is picked up by voting. It also gives an idea about the importance of the features.

The *naive Bayes Classifier* is known as the probability-based classifier. It will give the probability of whether the test data belongs to a class or not rather than specifying the label of the class to which it belongs. It is based on the Bayes theorem.

The *K-Nearest Neighbour Classifier (KNN)* is another classification algorithm that is often called a lazy learner machine learning algorithm since it does not consider training data for learning; instead, it uses them for computing the similarity with the new data points, and it places the new data points into the category which is most similar to the available ones.

All these eight machine learning algorithms have been implemented with the help of sklearn [24] on the previous curated training and test data as stated in the earlier section, and the performance accuracy of the best models is estimated.

(e) Prediction of novel COVID-19 human drug targets

The top three machine learning model predictors out of eight are now used to predict the COVID-19 human drug targets from the remaining 204,871 human proteins. The predicted COVID-19 human drug targets are further validated with the other COVID-19 human drug targets detected through several in-silico and in-vitro methodologies as specified in various works of literature [1,33,34,35,36]. A significant overlap of the predicted targets with the others has been observed, which motivates us to analyze those COVID-19 human drug targets further, which are not overlapped/matched. These might evolve as novel potential COVID-19 human drug targets if they relate to COVID-19 drugs or drug target pathways.

(f) Drug-Repurposing Study of novel COVID-19 human drug targets

The unmatched COVID-19 human drug targets now serve as an input to COVID19Db [37] to detect any possible connection with existing COVID-19 repurposed drugs. COVID19Db has an inbuilt drug discovery web server. This tool [37] initially led to the integration of the drug–target–pathway interactions from two central resources: (1) KEGG Pathway [21] resources and (2) DrugCentral [38]. Once the integration is done, the drug discovery tool is implemented with detailed results. The tool also generates PubMed database links to support the mapped drugs with corresponding drug targets [37]. This tool fetches disease pathway analysis, i.e., the number of targets, pathways, and target-pathway interactions, along with COVID-19-supported PubMed shreds of evidence for these unmatched COVID-19 human drug targets. Once the information is fetched, these novel COVID-19 human drug targets are further validated by docking studies in the next section.

(g) Docking study of the repurposed drugs detected through novel COVID-19 human drug targets

Molecular docking is an intrinsic requirement for any drug discovery [39]. With the help of DockCoV2 [40], a docking study is done on the repurposed drugs generated from the COVID19Db [37] for the novel COVID-19 human drug targets, as discussed earlier. DockCoV2 uses AutoDock Vina (version 1.1.2) [41] as its core docking utility. Docking analyses of the repurposed drugs have been performed with six COVID-19 proteins, including spike protein [42], 3CLpro [43], PLpro [44], RdRp [45], N protein [46], and ACE2 [47]. The study shows that the repurposed drugs might be the potential contenders for COVID-19.

## 3. Results & Discussion

This section can be broadly classified into two categories: 1) Performance Analysis of the Machine Learning Models used in ML-DTD: In this category, the results of the performance level of the models of ML-DTD will be discussed 2) Detection and Validation of novel COVID-19 Human Drug Targets: In this second category, the procedure of detecting novel human drug targets and their corresponding validation will be highlighted.

### 3.1. Performance Analysis of the Machine Learning Models Used in ML-DTD

**Figure 3:**
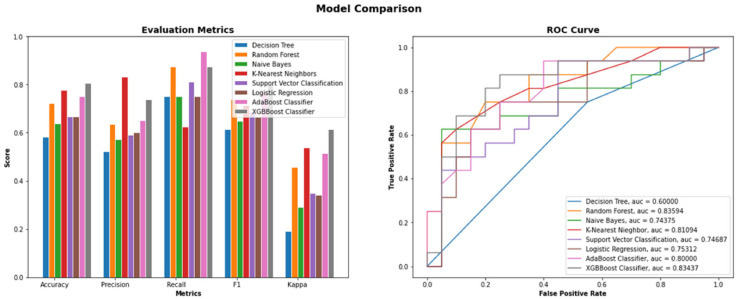
Comparison of Accuracy, Precision, Recall, F1-Score, Cohens Kappa Score, and AUC of the proposed ML-DTD.

In this section, the empirical results of the proposed machine learning methodology have been discussed. The positive and negative samples are formed from the TTD and human datasets. Eight machine learning models have been implemented on this curated dataset after dividing it into 80% training and 20% test set. The efficiency of the models has been estimated in terms of Accuracy, Precision, Recall, F1-Score, Cohens Kappa Score [48], and Area Under Curve [49], which is reported in Table 2 and Figure 3. XGBBoost Classifier obtained the best accuracy score of 0.80. The KNN and Random Forest models stand second and third by obtaining a score of 0.77 and 0.72, respectively.

In contrast, comparing the corresponding F1-Score, the top three model rankers remain the same, but their order changes slightly. XGBBoost Classifier holds its first position, while KNN and Random Forest model interchanges their position by obtaining an F1 score of 0.79, 0.71, and 0.73, respectively. The area under the curve (AUC) visually gives a more accurate representation for the prediction of COVID-19 human drug targets since it is calculated based on each model’s Receiver Operating Characteristic curve (ROC curve) to highlight the work’s quality. Figure 3 represents the eight machine learning models’ ROC and Area Under Curve (AUC). XGBBoost and Random Forest models predict the highest value in the AUC = 0.83 for the curated dataset, while KNN stands second with AUC = 0.81, respectively.

### 3.2. Detection and Validation of Novel COVID-19 Human Drug Targets

The three top ranking models, i.e., XGBBoost, KNN, and Random Forest models, as detected in the earlier stage, are selected and used to predict the novel COVID-19 targets from the human proteome consisting of 204,871 human proteins, which is obtained after removing the proteins involved in our curated dataset from the initial interactome consisting of 204,961 reviewed human proteins. XGBBoost, KNN, and Random Forest models successfully predict 3814, 1687, and 4144 COVID-19 novel human drug targets in the human proteome.

**Figure 4:**
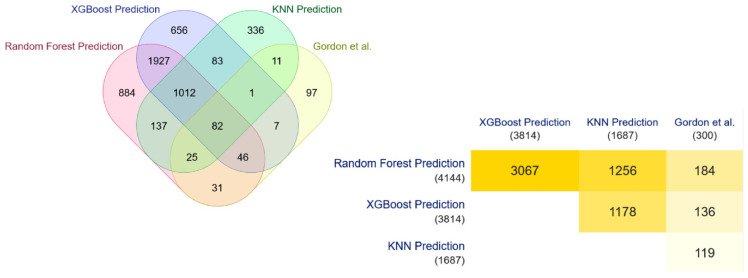
Overlapping of ML-DTD and Gordon et al. predicted COVID-19 human drug targets.

**Figure 5:**
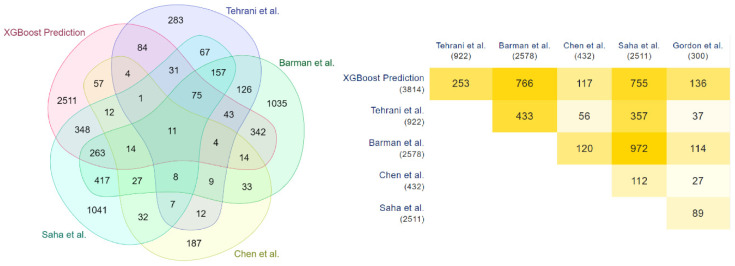
Overlapping of XGBoost and other methods predicted COVID-19 human drug targets.

**Figure 6:**
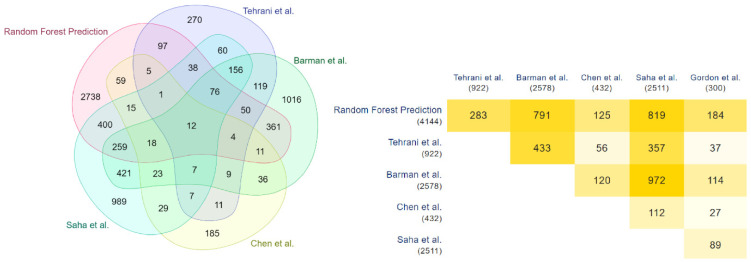
Overlapping of Random Forest and other methods predicted COVID-19 human drug targets.

**Figure 7:**
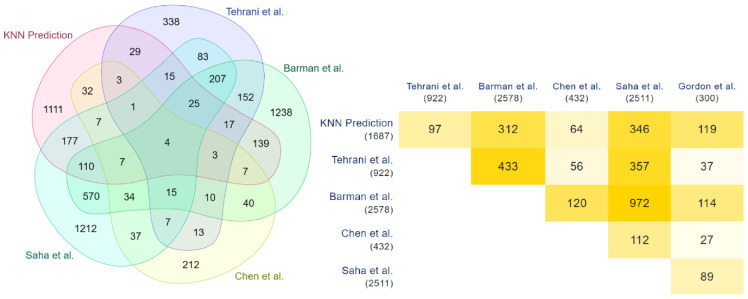
Overlapping of KNN and other methods predicted COVID-19 human drug targets.

These results are validated with the COVID-19 human drug targets detected through the in-vitro methodologies of Gordon et al. [35]. Significant overlap is observed in this validation, highlighted through the Venn diagram and the minimal triangular matrix in Figure 4. All the Venn diagrams and minimal triangular matrix are generated through the multi-list comparator tool of molbiotool [50]. Further analyses have been carried out, and the prediction of each model has been compared with the other in-silico methodologies of Tehrani et al. [36], Barman et al. [1], Chen et al. [33], and Saha et al. [34]. The results are highlighted in Figure 5, Figure 6 and Figure 7.

**Figure 8:**
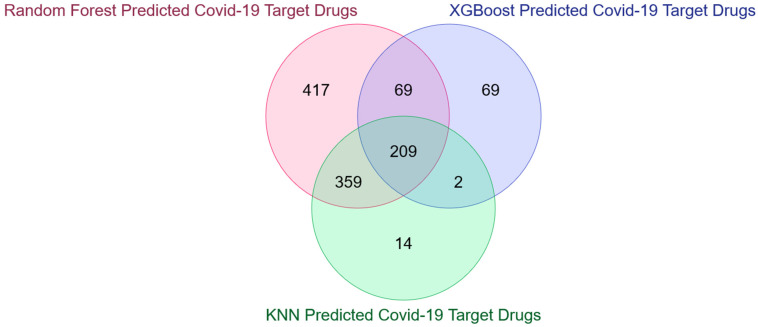
Overlap of COVID-19Db-derived potential COVID-19 drugs for the unmatched/novel targets predicted by the three top-performing models.

**Figure 9:**
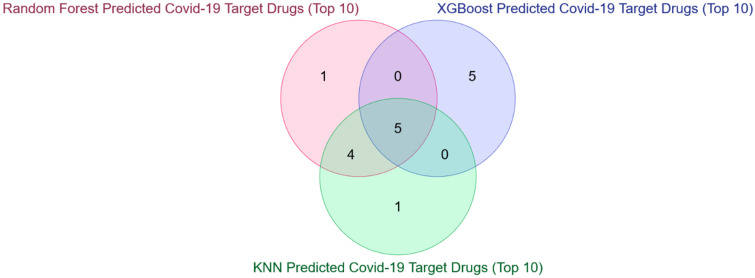
Overlap of top ten COVID-19Db-derived potential COVID-19 drugs for the unmatched/novel targets predicted by the three top-performing models.

The above results highlighted that some COVID-19 human drug targets are not overlapped with the existing targets, like 1070 in the case of KNN, 2680 in the case of RF, and 2468 in the case of the XGBoost model. These unmatched COVID-19 human drug targets for each model are then uploaded separately in COVID19Db [37], which gives two detailed results of the drugs associated with these targets: (1) Drug Target Pathway Interactions and (2) Number of target-pathway interactions of the potential actionable drugs along with the PubMed link of supporting shreds of evidence for COVID-19 (please see Supplementary Table S1 for XGBoost, Table S2 for RF and Table S3 for KNN). These potential drugs from each of the three models are collected, and it has been observed that there is an overlap of 209 drugs (please see Figure 8). Information for only these 209 drugs is retained for each of the three models from the above two results. These are then sorted in descending order based on the number of target-pathway interactions. The top ten drugs are selected from each model, and analyses have been made to see the possibility of overlap. The result is highlighted in Figure 9, which shows a common overlap of 5 repurposed COVID-19 drugs, and they are: (1) Bosutinib, (2) Crizotinib, (3) Midostaurin, (4) Nintedanib, and Sunitinib (please see Table 3).

**Figure 10:**
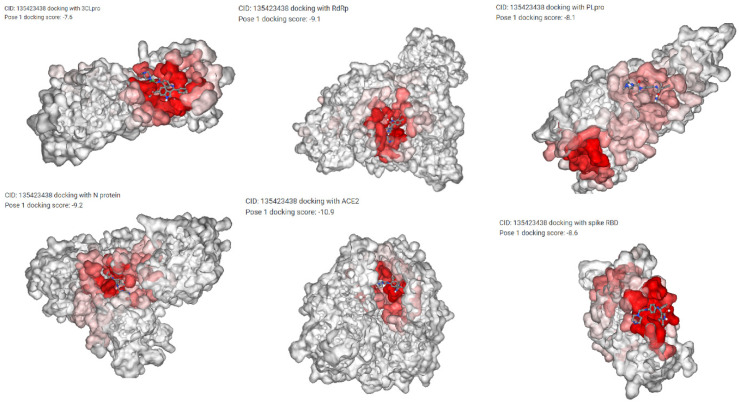
Best poses results of Docking of Nintedanib (CID: 135423438) with spike protein, 3CLpro, PLpro, RdRp, N protein, and ACE2.

All these 5 drugs are also docked with six COVID-19 proteins, including spike protein [42], 3CLpro [43], PLpro [44], RdRp [45], N protein [46], and ACE2 [47] by DockCoV2 [40]. DockCoV2 generates the docking score for each of them, highlighted in Table 4, where more negative scores indicate a strong binding of a protein-ligand complex. The generated scores are quite satisfactory for these five drugs. The best poses for each of them are displayed in Table 5. Few samples of best poses of Nintedanib with the other COVID-19 proteins are also highlighted in Figure 10. So, both COVID19Db and DockCoV2 prove that the unmatched targets (1070 in the case of KNN, 2680 in the case of RF, and 2468 in the case of the XGBoost model) can be treated as the novel COVID-19 human drug targets (please see Supplementary Table S4) since they have a good docking score with the COVID-19 proteins as well as they are linked with the COVID-19 repurposed drugs through target pathway analysis as discussed earlier.

## 4. Conclusions

In this paper, ML-DTD is proposed for identifying novel COVID-19 human drug targets, which are initially tested on a curated dataset of positive samples (obtained from the TTD dataset) and negative samples (formed from reviewed human proteome). PPIN features, protein sequence features, and GSEA ranking are used for this purpose. Then the best performing models are used to predict COVID-19 human drug targets in the remaining human proteome other than the curated dataset. A significant overlap has been found between the predicted and existing targets obtained from various existing works of literature. The non-overlapping targets are further analyzed and validated through COVID19Db and DockCoV2, highlighting that these unmatched targets can be the novel COVID-19 human drug targets. The accuracy of the proposed methodology can be further improved by including the protein domain and other allied features. Besides, UniProt and TTD do not include any strain specific annotations. So, other data sources to extract strain specific protein variants needs to be explored to prioritize drugs against evolving strains of SARS-CoV-2. Currently, this methodology is only used for COVID-19, which can be extended to other diseases in our future works.

## Figures and Tables

**Table 1:** Top twenty features selected by the SelectKBest module. SelectKBest module assigns a score to each feature which is then used for ranking. Features with lower rank are eliminated while higher ranking features are considered the most significant ones.

Sl. No.	Specifications/Features	SelectKBest Score
1	GO (BP) Rank2	7.62
2	GO (MF) Rank1	6.73
3	GO (BP) Rank1	5.25
4	GO (MF) Rank2	3.94
5	GO (CC) Rank1	3.34
6	Reactome Rank2	2.89
7	GO (CC) Rank2	2.82
8	Reactome Rank1	1.61
9	KEGG Rank1	1.59
10	KEGG Rank2	1.27
11	Betweenness	1.14
12	Tiny (PCP_TN)	0.72
13	Degree	0.6
14	Network	0.56
15	Sulphur Content (PCP_SC)	0.35
16	Solvent Accessibility (Intermediate) (PCP_SA_IN)	0.32
17	Cyclic (PCP_CY)	0.25
18	Small (PCP_SM)	0.21
19	Subgraph	0.212
20	Aromaticity (PCP_AR)	0.18

**Table 2:** The efficiency of the models used in ML-DTD. ML-DTD uses six parameters: Accuracy, Precision, Recall, F1-Score, Cohens Kappa Score, and Area Under Curve (AUC) to estimate the overall performance.

Sl. No.	Models	Accuracy	Precision	Recall	F1-Score	Cohens Kappa Score	Area under Curve
1	XGBBoost	0.80	0.73	0.87	0.79	0.61	0.83
2	AdaBoost	0.75	0.65	0.93	0.76	0.51	0.79
3	LR	0.66	0.60	0.75	0.66	0.34	0.75
4	SVM	0.66	0.59	0.81	0.68	0.34	0.74
5	Decision Tree	0.58	0.52	0.75	0.61	0.19	0.60
6	Random Forest	0.72	0.63	0.87	0.73	0.45	0.83
7	Naive Bayes	0.63	0.57	0.75	0.64	0.29	0.74
8	KNN	0.77	0.83	0.62	0.71	0.53	0.81

**Table 3:** Number of target-pathway interactions of the top five potential actionable COVID-19 repurposed drugs. COVID19Db maps these detected top 5 drugs with the disease pathway and generates the number of targets, pathways, and target-pathway interactions. It also highlights the PubMed literature links as evidence that these top 5 drugs are already associated with or recommended for the possible treatment of COVID-19 in other in-silico or in-vitro research methodologies.

Drug Name	The Number of Target-Pathway Interactions	The Number of Targets	The Number of Pathways	Evidence for COVID-19
Sunitinib	91	24	44	Link to PubMed
Bosutinib	66	20	43	Link to PubMed
Crizotinib	35	15	25	Link to PubMed
Midostaurin	47	6	39	Link to PubMed
Nintedanib	24	6	22	Link to PubMed

**Table 4:** Docking scores of top five potential actionable COVID-19 repurposed drugs with COVID-19 protein structures. The top five potential actionable COVID-19 repurposed drugs are also docked with the available COVID-19 proteases like 3CLpro, RdRp, PLpro, N, ACE2, and Spike RBD by DockCoV2. The docking score represents how well these drugs can be docked with COVID-19 proteases. More negative value highlights a good docking that represents that the drug and protease can dock well with each other by using less energy.

COVID-19 Structure Details		Predicted Drugs Docking Score by DockCoV2				
Structure Name	Structure ID	Bosutinib	Crizotinib	Midostaurin	Nintedanib	Sunitinib
3C-like protease	3CLpro	−7	−7.4	−8.5	−7.6	−7.1
RNA-dependent RNA polymerase	RdRp	−8.4	−7.9	−8.9	−9.1	−7.6
Papain-like protease	PLpro	−7.7	−7.3	−8.8	−8.1	−6.9
nucleocapsid protein	N	−9	−9.5	−10.1	−9.2	−8.8
ACE2 Receptor Protein	ACE2	−8.8	−9.2	−14.5	−10.9	−8
Spike Protein	Spike RBD	−7	−7	−8.8	−8.6	−8.3

**Table 5:** Best pose Links of the docking of top five potential actionable COVID-19 repurposed drugs with COVID-19 protein structures. DockCoV2 also generates the best docking pose for each of the top five detected potential COVID-19 drugs and COVID-19 proteases, the link of which is hyperlinked in the table below.

	Predicted Drugs Best Pose by DockCoV2				
Structure ID	Bosutinib	Crizotinib	Midostaurin	Nintedanib	Sunitinib
3CLpro	Best Pose Link	Best Pose Link	Best Pose Link	Best Pose Link	Best Pose Link
RdRp	Best Pose Link	Best Pose Link	Best Pose Link	Best Pose Link	Best Pose Link
PLpro	Best Pose Link	Best Pose Link	Best Pose Link	Best Pose Link	Best Pose Link
N	Best Pose Link	Best Pose Link	Best Pose Link	Best Pose Link	Best Pose Link
ACE2	Best Pose Link	Best Pose Link	Best Pose Link	Best Pose Link	Best Pose Link
Spike RBD	Best Pose Link	Best Pose Link	Best Pose Link	Best Pose Link	Best Pose Link

## Data Availability

Not applicable.

## Supplementary Materials

The following supporting information can be downloaded at: https://www.mdpi.com/article/10.3390/vaccines10101643/s1, Table S1: Drug Target Pathway Interaction of 2468 unmatched targets by XGBoost (generated by Covid-19Db); Table S2: Drug Target Pathway Interaction of 2680 unmatched targets by RF (generated by Covid-19Db); Table S3: Drug Target Pathway Interaction of 1070 unmatched targets by KNN (generated by Covid-19Db); Table S4: NOVEL COVID-19 Human Targets predicted by XGBoost, KNN and RF.
